# Net2Brain: a toolbox to compare artificial vision models with human brain responses

**DOI:** 10.3389/fninf.2025.1515873

**Published:** 2025-05-06

**Authors:** Domenic Bersch, Martina G. Vilas, Sari Saba-Sadiya, Timothy Schaumlöffel, Kshitij Dwivedi, Christina Sartzetaki, Radoslaw M. Cichy, Gemma Roig

**Affiliations:** ^1^Department of Computer Science, Goethe Universität, Frankfurt am Main, Germany; ^2^The Hessian Center for Artificial Intelligence, Darmstadt, Germany; ^3^Ernst Strüngmann Institute for Neuroscience, Frankfurt am Main, Germany; ^4^Informatics Institute, University of Amsterdam, Amsterdam, Netherlands; ^5^Department of Education and Psychology, Freie Universität at Berlin, Berlin, Germany; ^6^Faculty of Philosophy, Berlin School of Mind and Brain, Berlin, Germany; ^7^Bernstein Center for Computational Neuroscience Berlin, Berlin, Germany

**Keywords:** cognitive neuroscience, deep neural networks, neuroimaging data analysis, artificial intelligence in neuroscience, toolbox, multimodal neural models

## Abstract

In cognitive neuroscience, the integration of deep neural networks (DNNs) with traditional neuroscientific analyses has significantly advanced our understanding of both biological neural processes and the functioning of DNNs. However, challenges remain in effectively comparing the representational spaces of artificial models and brain data, particularly due to the growing variety of models and the specific demands of neuroimaging research. To address these challenges, we present Net2Brain, a Python-based toolbox that provides an end-to-end pipeline for incorporating DNNs into neuroscience research, encompassing dataset download, a large selection of models, feature extraction, evaluation, and visualization. Net2Brain provides functionalities in four key areas. First, it offers access to over 600 DNNs trained on diverse tasks across multiple modalities, including vision, language, audio, and multimodal data, organized through a carefully structured taxonomy. Second, it provides a streamlined API for downloading and handling popular neuroscience datasets, such as the NSD and THINGS dataset, allowing researchers to easily access corresponding brain data. Third, Net2Brain facilitates a wide range of analysis options, including feature extraction, representational similarity analysis (RSA), and linear encoding, while also supporting advanced techniques like variance partitioning and searchlight analysis. Finally, the toolbox integrates seamlessly with other established open source libraries, enhancing interoperability and promoting collaborative research. By simplifying model selection, data processing, and evaluation, Net2Brain empowers researchers to conduct more robust, flexible, and reproducible investigations of the relationships between artificial and biological neural representations.

## 1 Introduction

Over the past decade, DNNs have emerged as a powerful class of computational tools in visual neuroscience. DNNs outperform other models in predicting brain activity during visual processing and have been instrumental in explaining and exploring the nature of visual brain functions (Yamins and DiCarlo, 2016; Kietzmann et al., 2019; Saxe et al., 2021; Cadieu et al., 2014; Khaligh-Razavi and Kriegeskorte, 2014; Yamins et al., 2014; Guclu and Van Gerven, 2015; Cichy et al., 2016; Zhuang et al., 2021). By comparing the predictive power of key DNN properties, such as model architecture, objective functions, and training regimes, researchers are advancing our understanding of the computational and functional characteristics of visual cortex regions (Dwivedi et al., 2021; Ratan Murty et al., 2021; Richards et al., 2019; Cichy and Kaiser, 2019; Doerig et al., 2022; Bakhtiari et al., 2021; Khaligh-Razavi and Kriegeskorte, 2014; Guclu and Van Gerven, 2015; Seeliger et al., 2018).

However, the rapid growth and accelerating use of DNNs in visual neuroscience pose significant challenges for individual researchers and the field as a whole. One key challenge is the continual evolution and rapidly expanding number of DNNs, presenting researchers with a very large set of experimental choices. This complicates the selection of appropriate models for specific research questions, as there is no systematic method to assess models beyond individual comparisons. Consequently, this makes it difficult to consolidate and compare findings from previous studies, limiting the integration, and generalization of results obtained with DNNs.

A second, related challenge is the absence of standardized protocols and criteria for selecting and predicting brain data. This issue spans all aspects of research with DNNs, from extracting their internal representations to how models are linked to brain activity and comparing model performance. As a result, researchers often develop individualized approaches and rely on custom code, which can negatively impact documentation, reproducibility and generalization of findings (Miłkowski et al., 2018).

To address these challenges, we introduce Net2Brain, an easy-to-use, end-to-end toolbox for bridging neuroscience with AI research. Net2Brain tackles the first challenge by systematically organizing over 600 models, simplifying the process of selecting and comparing DNNs. It addresses the second challenge by offering standardized yet flexible procedures for all experimental steps, eliminating the need for custom coding. This streamlines access to neuroscience datasets, facilitates preprocessing, and provides widely used analysis options, along with tools for data analysis and visualization. With these features, Net2Brain supports and accelerates research at the intersection of artificial and biological neural networks in a sustainable, robust, and reproducible manner.

## 2 Related work

Several toolboxes are already available carrying out research at the intersection of neuroscience and AI. Below, we briefly characterize these toolboxes and demonstrate how Net2Brain complements them (see Appendix Table 1 for a structured comparison).

The **RSAToolbox** offers a comprehensive set of functions for comparing the representational spaces of different systems, including those of brains and DNNs. Starting from the internal representations of DNNs provided by the user, it facilitates all subsequent steps of analysis and statistics through RSA (Nili et al., 2014; Kriegeskorte, 2008). Net2Brain incorporates key features of the RSAToolbox, such as RSA and weighted RSA, while extending its functionality with additional tools like linear encoding and variance partitioning analysis. Moreover, Net2Brain integrates feature extraction capabilities, allowing users to directly interact with both the RSAToolbox and other analysis pipelines within a broader experimental framework.

**THINGSvision** extracts activations from a wide range of pre-trained vision-related DNNs for user-provided images, enabling comparisons between DNNs and the THINGS brain and behavioral datasets (Hebart et al., 2019). Net2Brain complements THINGSvision by offering an end-to-end pipeline that includes feature extraction from a large set of models. In addition, it provides easy access to other brain datasets, such as NSD (Gifford et al., 2023) and BOLDMoments (Lahner et al., 2024), and extends to DNNs beyond vision, including multimodal, audio, and large language models.

**BrainScore** is an online benchmarking platform (Schrimpf et al., 2018, 2020) where users submit models to be compared against a set of brain activations, generating a score that reflects how well the model's activations predict brain activity. BrainScore primarily uses encoding models and focuses on non-human primate visual brain data. Net2Brain complements BrainScore by streamlining access to human visual brain datasets and providing a diverse range of evaluation functions commonly used in the literature, while also enabling users to prepare and submit research to BrainScore.

**The Algonauts Project** and the **Sensorium competition** are recurring online challenges that invite participants to predict human and non-human brain data, typically recorded in visual experimental settings (Cichy et al., 2019; Lahner et al., 2024; Gifford et al., 2023; Willeke et al., 2022; Turishcheva et al., 2024). Participants submit brain data predictions, which are tested against held-out empirical data, with the best prediction winning the challenge. Net2Brain complements these efforts by offering offline, user-defined flexibility in terms of experimental parameters at all stages.

## 3 The Net2Brain toolbox: overview and core functionality

**Figure 1:**
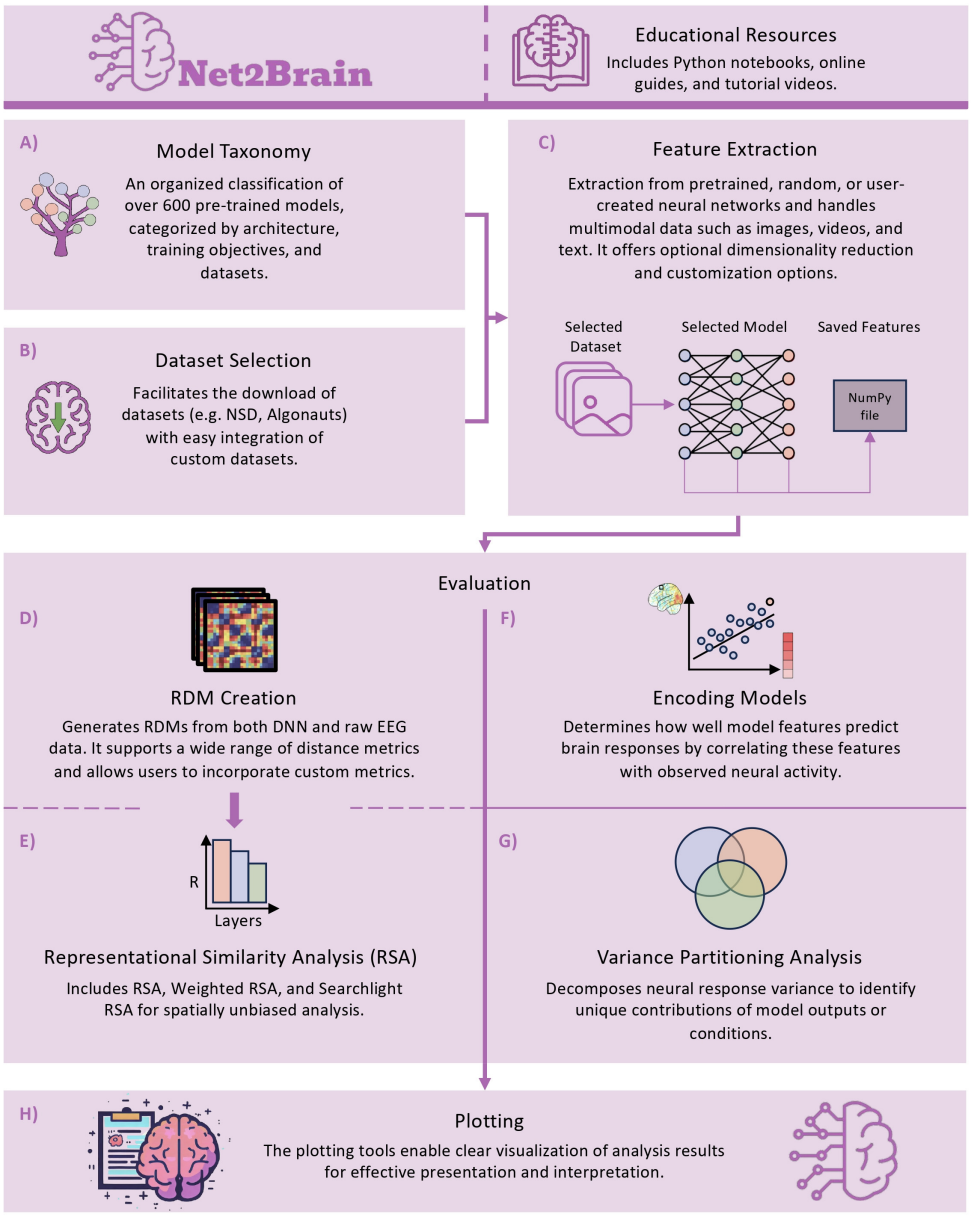
The complete pipeline of Net2Brain, divided by core functionalities. **(A)** Model taxonomy, helping users select models based on architecture and task. **(B)** Dataset downloads, providing access to key neuroscience datasets. **(C)** Feature extraction from model layers. **(D, E)** RDM creation, generating Representational Dissimilarity Matrices for RSA, including Weighted RSA and Searchlight analysis. **(F, G)** Encoding models and variance partitioning analysis for evaluating model performance in predicting brain responses. **(H)** Visualization of results through the plotting module.

Net2Brain (see Figure 1) is a Python-based, end-to-end, open-source toolbox designed to relate DNNs to human brain data that is publicly available on GitHub.^1^ The repository includes a well-documented codebase and a comprehensive collection of Python notebooks that provide practical, step-by-step tutorials for utilizing the toolbox's various features as well as notebooks demonstrating how to replicate previous studies using Net2Brain. In addition, the Net2Brain documentation website offers multiple resources, including tutorial videos and extended guides covering everything from basic setup to advanced applications. Net2Brain follows a modular and interoperable design philosophy, enabling seamless integration with other toolboxes, such as THINGSvision and the RSAToolbox. This approach allows researchers to extend existing workflows with Net2Brain's functionalities or flexibly build new workflows by combining different sources.

The end-to-end pipeline of Net2Brain consists of six main components (see Figure 1 for an overview): First, the taxonomy module (Figure 1A) assists in selecting appropriate models from a diverse set of over 600 models spanning various modalities and architectures. Second, the dataset selection module (Figure 1B) provides access to multiple human brain datasets. Third, the feature extraction module (Figure 1C) extracts internal model representations. Fourth, the RDM creation module (Figure 1D) generates representational dissimilarity matrices (RDMs) from both model activations and brain data when performing RSA. Fifth, the evaluation module relates brain activations to model representations using RSA or encoding models, enriched by advanced comparative techniques like variance partitioning (Figures 1E–G). Finally, the plotting module visualizes the results (Figure 1H). For details on runtime and memory usage across different architectures and computing environments, see Appendix Table 2. In the following sections, we will describe each module in more detail.

### 3.1 Model taxonomy module

The model taxonomy module (Figure 1A) is designed to help researchers select the most suitable models from a pool of over 600 DNNs, based on their research objectives (see Appendix Tables 3, 4). This set includes both pre-trained and randomly initialized models. The taxonomy organizes models based on various attributes, including architecture types (e.g., Convolutional Neural Networks, Large Language Models), training tasks (e.g., image classification, video classification), datasets used for training [e.g., ImageNet (Russakovsky et al., 2015), COCO (Lin et al., 2014)], and training methods [e.g., supervised vs. self-supervised techniques like SimCLR (Chen et al., 2020) and MoCo He et al. (2020)].

The module also includes functions for targeted searches. For example, the model_like_name() function helps researchers find models similar to a term of choice, while the find_model_by_custom() function enables custom-defined searches based on a combination of attributes. These functions return a list of suitable models along with their labels, allowing the user to select them during the feature extraction step.

### 3.2 Dataset selection module

The dataset selection module of Net2Brain (Figure 1B) loads preprocessed brain data from our database along with the corresponding stimuli that elicited the brain responses, allowing for the extraction of DNN activations by processing the same stimuli used in the neuroscience experiment. The module readily accommodates custom datasets, enabling researchers to load their own data into Net2Brain.

Net2Brain streamlines access to a collection of popular datasets widely used in the cognitive neuroscience research community, each including its own set of corresponding regions of interest (ROIs) (see Appendix Table 5). These integrated datasets are based on healthy participants and include the NSD dataset, formatted for the Algonauts Challenge 2023 (Gifford et al., 2023), which contains high-resolution fMRI responses to tens of thousands of natural scenes; an additional NSD subset containing the 1,000 stimuli viewed by all eight subjects (Allen et al., 2022); the THINGS fMRI-Dataset (Hebart et al., 2023, 2019), featuring brain responses to images of everyday objects; the Algonauts 2019 dataset, which focuses on predicting brain activity in response to object recognition using fMRI and MEG data (Cichy et al., 2019); the BOLD Moments Dataset from Algonauts 2021, capturing fMRI responses to short naturalistic video clips to study dynamic visual perception (Lahner et al., 2024) and a set of complex natural scenes used to study navigational affordances in the human visual system (Bonner and Epstein, 2017). In the coming months, we plan to expand this collection further by incorporating datasets from the cNeuromod project. For the NSD dataset, Net2Brain offers enhanced functionalities that further facilitate analysis. It automates the download of segmentation masks and COCO captions, provides ID translations between NSD and COCO, and offers additional visualization options for images and segmentation masks.

### 3.3 Feature extraction module

The feature extraction module (Figure 1C) enables the extraction of internal representations from models pre-trained for different tasks, randomly initialized networks, or user-provided models. Net2Brain also suggests a set of summarizing layers for extraction, while also allowing researchers the flexibility to select any layer of interest. In addition to its core functionality, the module includes dimensionality reduction techniques (e.g., Principal Component Analysis (PCA), Sparse Random Projection) to efficiently manage and analyze high-dimensional model activation data.

### 3.4 RDM creation module

The RDM creation module (Figure 1D) generates RDMs from both the brain data loaded by the dataset selection module and the model activations passed from the feature extraction module.

RDMs (Kriegeskorte, 2008) are a technique that abstracts incommensurable multivariate measurement spaces–such as those from DNNs and brain measurements–into a common similarity space. This is achieved by computing RDMs, which are 2D matrices that summarize the representational geometry of a measurement space. RDMs are indexed by rows and columns representing the experimental conditions compared, and they store the dissimilarity between activation patterns associated with these conditions. When the same stimulus set is used across different measurement spaces, the resulting RDMs are of equal format and can be directly compared.

To calculate dissimilarity between activation patterns, the RDM creation module offers a range of common distance metrics (e.g., pearson, cosine, euclidean) and allows users to define custom metrics as needed. Hardware-accelerated computing via GPU and matrix chunking techniques enable efficient RDM computation even for large activation datasets.

### 3.5 Evaluation module

The evaluation module provides tools for linking model activations to brain responses using either RSA or encoding techniques. RSA relies on comparing RDMs between models and brain data, while encoding techniques directly map raw model features to brain responses using a regression model.

For RSA (Kriegeskorte, 2008) (see Figure 1E), the module compares the model and brain RDMs generated by the RDM creation module. By default, the standard RSA uses Pearson correlation distance to measure the similarity between the RDMs, and thus the representational spaces of both systems, though users can define other metrics based on their research needs. The module offers flexible correlation averaging methods across subjects, supporting both squared and direct correlation averaging. Additionally, the module supports weighted RSA, an advanced form of RSA that adjusts the influence of data points in the computation (Kriegeskorte, 2008). The module also enables spatially unbiased fMRI searchlight analysis (Kriegeskorte et al., 2006; Haynes and Rees, 2005).

For encoding models, the module offers two related options. The first option is linear regression, using model activations to predict brain activity patterns (Yamins et al., 2014; Naselaris et al., 2011) (see Figure 1F), which includes Ridge Regression and stacked encoding (Lin et al., 2024). Stacked encoding combines multiple feature spaces through a weighted linear combination. The module also implements veRSA (voxelwise encoding RSA), combining encoding models with representational similarity analysis to evaluate alignment between predicted and actual voxel patterns (Khaligh-Razavi et al., 2017; Conwell et al., 2024). The similarity between predicted and actual brain data provides a measure of how well the model predicts brain activity. The second option is variance partitioning analysis (Legendre, 2008) (see Figure 1G), and structured variance partitioning (Lin et al., 2024), which decomposes the variance in brain data through multiple linear regression. These methods attribute variance to different sources, such as various model outputs or experimental conditions. While traditional variance partitioning helps identify which aspects of the model align most closely with brain data, structured variance partitioning leverages known relationships between features during hypothesis testing, allowing for targeted questions about similarity between feature spaces and brain regions even when feature spaces are correlated.

The module also includes Centered Kernel Alignment (CKA) (Kornblith et al., 2019), which measures similarity across high-dimensional spaces. Additionally, it supports distributional comparisons, such as Jensen-Shannon Divergence and Wasserstein Distance, which assess the statistical alignment between representational distributions.

In all cases, the evaluation module outputs results in a standardized format, ensuring integration with the visualization and plotting module.

### 3.6 Visualization module

Net2Brain includes plotting functionalities (see Figure 1H) that enable users to visualize evaluation results in a publication-ready format. Users can create bar plots to display correlation values from the evaluation module, including noise ceilings, statistical significance, and optionally pairwise significance for all model layers to provide a comprehensive overview. Alternatively, users can opt for a condensed view that highlights only the best-performing layer, which is especially useful when analyzing multiple models and regions of interest. For time series data, such as EEG, similar options are available through line plots.

## 4 Walkthrough through an example application

To demonstrate how Net2Brain works, we present a walk-through of a detailed case study. In this example, we focus on the predictive capabilities of various Large Language Models (LLMs) and vision transformers in modeling activity in the human visual cortex. This is a timely topic in visual neuroscience, as LLMs have recently and unexpectedly emerged as strong models for high-level visual cortex activity - an alternative to the vision-centric models traditionally used (Doerig et al., 2022; Toneva and Wehbe, 2019; Muttenthaler et al., 2023; Schwartz et al., 2019). Our objective with these experiments is to evaluate the performance of different DNNs in predicting visual brain activity, and explore which aspects of these artificial models influence their predictive accuracy.

We chose to replicate a well-established finding in the neuroscience literature to demonstrate the robustness and accuracy of Net2Brain's methods. By showing that Net2Brain can reproduce reliable results, we aim to encourage both replication and novel experimental designs using the toolbox.

To support this case study, we provide a step-by-step tutorial notebook, available in the "notebooks" directory of the Net2Brain repository, titled "Net2Brain Linear Encoding". This tutorial guides users through each step of the process, from the initial model selection using Net2Brain's model taxonomy to final result visualization and includes detailed implementation information for those seeking a comprehensive guide.

### 4.1 Step 1: model selection using Net2Brain's taxonomy

**Figure 2:**
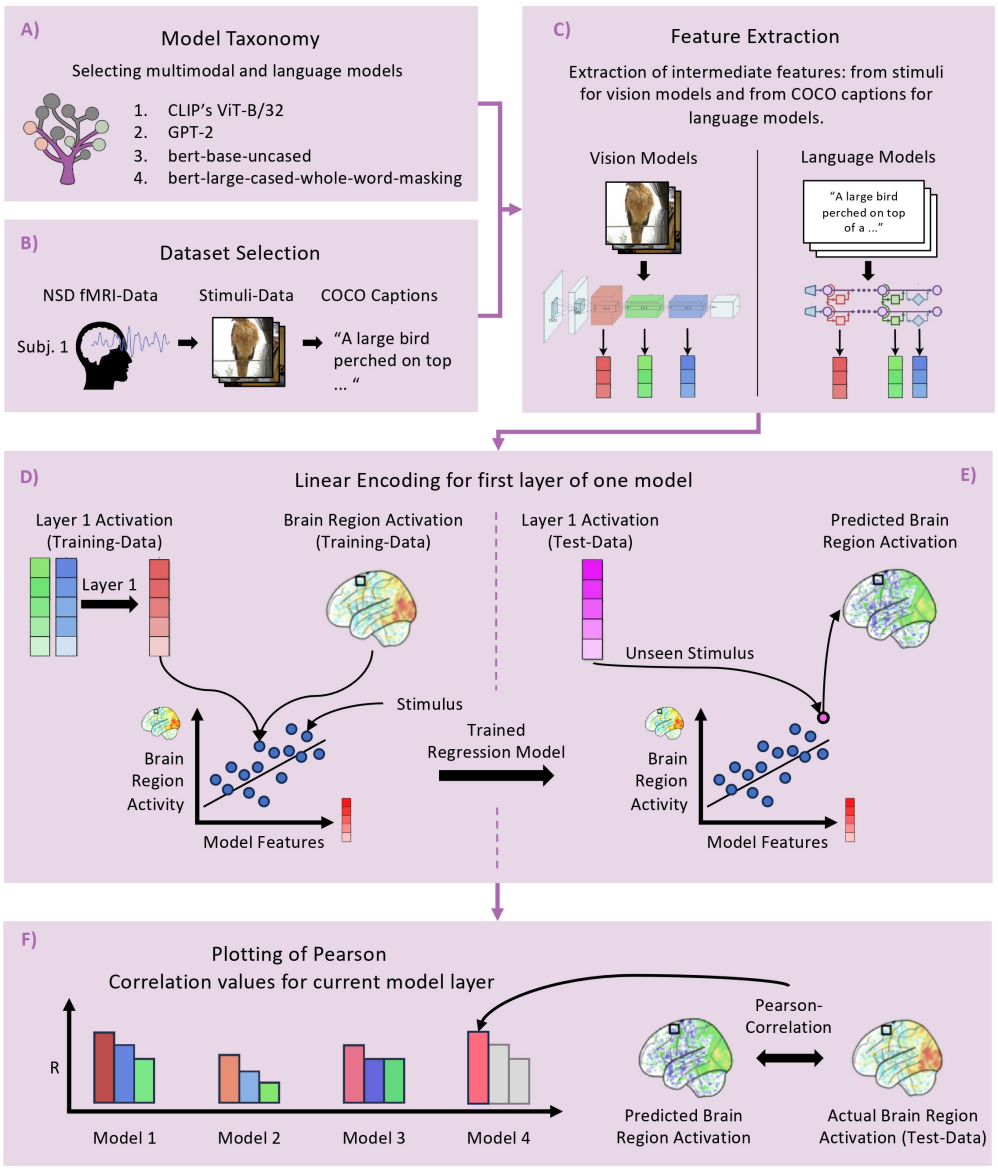
Workflow of the example experiment conducted using Net2Brain. **(A)** Model Taxonomy, highlighting the selection of multimodal and language models. **(B)** Dataset Selection: involves retrieving the NSD fMRI dataset, focusing on brain activations from subject 1. **(C)** Feature Extraction: activations are extracted from vision and language models using images and COCO captions [with visualization from Meng et al. (2022)]. **(D)** Linear Encoding: a model is trained to predict brain activity from each DNN layer across brain regions. **(E)** Demonstration of applying the trained models to unseen data, comparing predicted brain activations with actual responses using Pearson correlation to assess predictive accuracy.

In the first step, we leverage Net2Brain's model taxonomy to select suitable models for our experiment, as shown in Figure 2A. By applying the taxonomy's filter functions, we generate a list of models that we further refine based on our experimental goals. Our objective is to evaluate the performance of different LLMs in predicting visual brain activity, so we focus on models that allow us to assess various aspects of LLM functionality.

We start by filtering for multimodal models that process both visual and textual inputs. This leads us to select CLIP's ViT-B/32 (Radford et al., 2021), a model equipped with dual encoders for images and captions. CLIP's dual-encoder architecture offers insights into how each encoder processes its respective input, allowing us to compare the contributions of visual and textual information in predicting brain activity. Next, we select two variants of the BERT encoder model that differ in their pre-training tasks: bert-base-uncased and bert-large-cased-whole-word-masking (Devlin et al., 2018). The key difference lies in their pre-training objectives: the bert-base-uncased model is trained to predict individual masked tokens, while the bert-large-cased-whole-word-masking model is trained to predict entire masked words, which may consist of multiple tokens. This comparison allows us to investigate how the different pre-training tasks, particularly whole word vs. subword prediction, affect brain activity prediction accuracy.

Finally, to explore a different architecture, we select GPT-2 (Radford et al., 2018), a transformer-based decoder model that generates text by predicting the next word in a sequence. Including GPT-2 enables us to assess whether its generative nature and distinct architecture affect its ability to predict visual brain activity compared to the encoder models chosen earlier.

### 4.2 Step 2: downloading the NSD dataset

In the second step (see Figure 2B), we identify and prepare the relevant dataset, with Net2Brain's dataset API facilitating access to a diverse array of options. For this experiment, we focus on the NSD dataset, a large collection of brain responses recorded using 7T fMRI across the cortex in response to a wide range of real world images selected from the COCO database, which includes captions, annotations, and segmentation masks. Due to its high quality and extensive number of conditions, this dataset is particularly well-suited for exploring the relationship between DNNs and the visual brain.

We use the fMRI data of subject 1 as preprocessed for the Algonauts Challenge 2023. This dataset consists of preprocessed fMRI responses projected onto a common cortical surface group template, focusing on a subset of cortical surface vertices in the visual cortex. Each voxel's activity was independently z-scored for each session, and responses were averaged across repeated presentations of the same stimuli. During the NSD experiment, each subject viewed approximately 10,000 distinct images, each presented three times, resulting in 30,000 image trials. The experiment spanned 40 scan sessions, with the final three sessions withheld for the test split of the Algonauts Project 2023 Challenge.

For this study, we have merged the fMRI data from both hemispheres and combined data from the ventral and dorsal pathways (V1–V3). The regions of interest for this experiment include the early visual cortex (V1, V2, V3, hV4), which is responsible for processing low-level visual features such as edges, orientation, motion, and spatial frequencies. The word form regions (VWFA-1, VWFA-2) specialize in recognizing written words and orthographic patterns, offering insights into text-related neural processing. The face-selective region (FFA-1) plays a key role in face recognition, while the scene-selective region (PPA) is known for its involvement in processing spatial layouts and scenes. These regions provide a comprehensive view of neural responses across different levels of visual and textual processing.

In addition to the fMRI data and stimulus images, we download captions associated with each image sample from the COCO database. As we focus on LLMs in this study, the captions will serve as the stimuli for which we will extract model activations during the feature extraction phase.

### 4.3 Step 3: feature extraction

After downloading the NSD dataset and selecting the appropriate models, we proceed to the feature extraction process (see Figure 2C) to obtain model activations. For this, we use the feature extraction module. We provide the path to the dataset and the names of the selected models as inputs to the feature extractor, which then extracts features from the predefined or user-specified layers of each model. For all models, we focus on the final layers of key blocks within the architectures.

Net2Brain stores the extracted activations for each model layer in individual numpy files. These files contain a dictionary where each image ID serves as a key, with the corresponding activations from that layer as the values. We repeat this extraction and storage process for all selected models. The resulting files will serve as the basis for the subsequent evaluation.

If we were using RSA, RDMs would be created from the activations using the RDM creation module before passing them to the evaluation module. In this example, however, we will use linear encoding to relate model activations to brain data. Therefore, we will use the raw activation values as inputs for the evaluation function.

### 4.4 Step 4: evaluation by linear encoding

To begin the evaluation process, we load the linear encoding module within Net2Brain, rather than the RSA or variance partitioning module (see Figures 2D, E). The evaluation is conducted separately for each layer of each model. We input the extracted activations, reduced via PCA to 100 components, along with the corresponding NSD brain data. The module splits the image and brain data into training and testing sets, using an 80/20 train-test split across three cross-validation folds. For each fold, a linear regression model from scikit-learn (Pedregosa et al., 2011) is trained on the training data to map model activations onto brain responses. It is then tested on the unseen test data. The predictive ability of the model is assessed by computing the Pearson correlation between the predicted and actual brain responses to the test data, providing a quantitative measure of model performance (see Figure 2F). This process is repeated for all selected models and layers. Upon completion, the linear encoding module returns a dataframe containing the results, which are ready to be visualized in the next step.

### 4.5 Step 5: visualizing results

**Figure 3:**
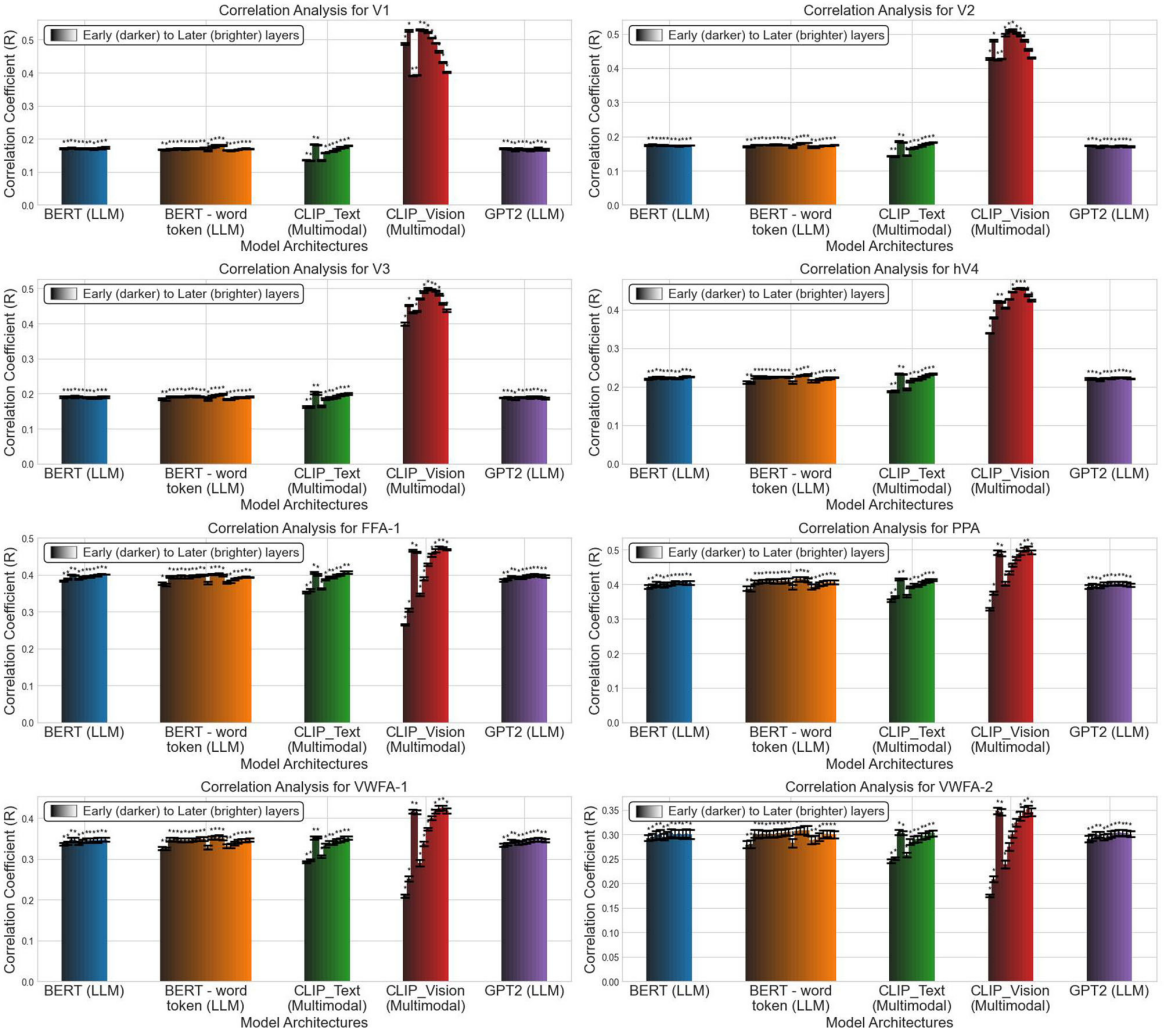
Visualization of Pearson correlation (R) across all model layers for each brain region of subject 1. Each color represents a distinct model, with darker hues indicating earlier layers and lighter hues representing later layers. Statistically significant results (*p* < 0.05) are marked with an asterisk. Each subplot corresponds to a specific region of interest, showing the correlation between model layers and brain regions. Regions include the early visual cortex (V1, V2, V3, hV4), face-selective region (FFA-1), scene-selective region (PPA), and word form regions (VWFA-1, VWFA-2). Model labels include “BERT” (11 layers), “BERT (word token)” (23 layers), “CLIP_Text”, “CLIP_Vision”, and ‘GPT2” (all with 11 layers).

After obtaining the dataframe from the Linear Encoding module, we use Net2Brain's plotting module to visualize the results. Figure 3 shows the predictive power of each layer for the investigated models across a set of brain regions: the early visual cortex, face-selective area FFA-1, parahippocampal place area PPA, and visual word form areas VWFA-1 and VWFA-2.

The visualizations reveal several key experimental observations. First, focusing on the visual encoder of the CLIP transformer architecture (red bars), we observe a hierarchical correspondence between the human brain and the model. Specifically, low-level visual regions are better explained by early model layers, while high-level visual regions are better explained by late model layers. This pattern aligns with previously observed findings (Cichy et al., 2016; Eickenberg et al., 2017; Guclu and Van Gerven, 2015), and suggests a parallel processing hierarchy in both human brains and DNNs.

Second, we find that language-related models (i.e., the text encoder of CLIP, the two BERT variants, and GPT-2) rival the visual transformer of CLIP in predicting brain activity in high-level visual areas. However, in low-level visual areas, the visual encoder of CLIP outperforms the language models. Between the two BERT variants, there is no significant difference in their performance across the brain regions. These results are consistent with prior research (Doerig et al., 2022; Haynes and Rees, 2005), suggesting that the high-level ventral visual cortex conducts complex semantic analyses of visual input, akin to the semantic content captured in captions describing the visual stimuli.

## 5 Discussion

In this paper, we introduced Net2Brain, a Python-based toolbox designed to facilitate the integration of DNNs with cognitive neuroscience research. Net2Brain promotes four key goals: (1) streamlining research, (2) fostering a collaborative and integrative research environment, (3) providing low-threshold access, and (4) increasing the reliability and generalizability of research. Below, we outline how Net2Brain contributes to each of these areas.

First, Net2Brain streamlines research by offering an end-to-end solution that includes a model taxonomy to guide the selection of appropriate models, along with feature extraction, evaluation, and visualization modules that automate the full workflow once parameters – such as model, dataset, metrics, and their finer settings – are manually specified. Second, it fosters a collaborative and integrative research environment through its modular design, enabling researchers to use Net2Brain in its entirety or to integrate specific components into their existing workflows. Third, it provides easy access to a broad range of models and offers automated access to key datasets, accelerating innovative and interdisciplinary research, particularly for junior researchers. By reducing technical barriers, Net2Brain allows researchers to focus more on scientific questions rather than computational complexities. Fourth, it enhances the reliability and generalizability of research by providing a standardized way to test multiple models, making it easier to replicate studies and assess the generalizability of results.

Net2Brain is designed to evolve and adapt to the needs of the research community. Ongoing development efforts aim to expand its functionalities by incorporating additional datasets, enhancing the processing of multimodal data and including video datasets. We are also introducing new evaluation metrics and expanding the toolbox's visualization capabilities to offer deeper insights. Additionally, more tutorials are being developed to help researchers effectively utilize these new features.

With its modular and adaptable design, we envision Net2Brain to be a highly valuable tool for researchers working at the intersection of DNNs and neuroscience. Net2Brain is expected to continuously evolve through community-driven use and contributions, ensuring it remains aligned with the needs of the scientific community and continues to expand its capabilities.

## 6 Limitations

Net2Brain provides researchers the freedom to select and combine models for their experiments without imposing restrictions. Although this flexibility encourages diverse neuroscientific approaches, it also requires users to ensure their experimental designs are conceptually sound. To support informed choices, however, the taxonomy module provides detailed insights into each model's training tasks, datasets, and methods, helping users understand the context and characteristics of their selected models.

While Net2Brain offers a robust set of evaluation metrics, it does not encompass every method currently available for comparing DNNs and brain data. However, its modular design allows users to integrate their own evaluation techniques seamlessly. By continuously incorporating feedback from the community, Net2Brain regularly expands its offerings, ensuring the toolbox evolves to meet diverse research needs.

Net2Brain does not include built-in visualization tools for projecting fMRI data onto brain surfaces, focusing instead on facilitating comparisons between DNNs and preprocessed brain data. Users can complement Net2Brain with specialized visualization tools like Pycortex (Gao et al., 2015) and Nilearn (Abraham et al., 2014).

The datasets provided through Net2Brain represent a carefully curated subset of those available in the neuroscience community, serving as a starting point for research and tutorials. While not exhaustive, this collection is designed to balance accessibility with functionality, and users can easily extend their analyses by incorporating additional datasets tailored to their specific needs.

While these limitations reflect the trade-offs necessary to balance flexibility and usability, Net2Brain's community-driven approach ensures that it will continue to address user needs and expand its capabilities over time.

## Data Availability

The original contributions presented in the study are included in the article/supplementary material, further inquiries can be directed to the corresponding author.

## Footnotes

^1^ https://github.com/cvai-roig-lab/Net2Brain
