# Supplementary material for: Net2Brain: a toolbox to compare artificial vision models with human brain responses
Source: Front Neuroinform. 2025 May 6;19:1515873. doi: 10.3389/fninf.2025.1515873 (PMC12089098; doi:10.3389/fninf.2025.1515873)
Supplement: Supplementary file 1 [file Table_1.pdf]

Supplementary Material

1 SUPPLEMENTARY DATA

Table S1. Comparison of Net2Brain with other neuroscience-AI frameworks and toolboxes

Toolbox	Available Datasets	Models	Brain-Model Alignment Methods	DNN Activation Extraction
RSAtoolbox	N/A	N/A	RSA, Weighted RSA, Various dissimilarity measures	No
BrainScore	Behavioral and neural benchmarks from humans and primates, consisting of 106 **Vision** and 33 **Language** benchmarks	N/A	Dataset specific	No
THINGSvision	THINGS Dataset, supports user-defined HDF5 datasets	**Vision** DNNs from various sources	RSA, CKA	Yes
Net2Brain	Algonauts 2019 datasets, Bold Moments, Algonauts 2023 Dataset (NSD) and 872-Subjects Subset, THINGS fMRI test-set, Bonner et al. 2017, supports user-defined datasets	Models including **Vision**, **Video**, **Language**, **Audio**, and **Multi-modal** DNNs, also accepts custom models	RSA, CKA, Linear and Stacked Encoding, Searchlight, Variance Partitioning, Structured Variance Partitioning	Yes

Table S2. Benchmarking the runtime and memory usage of an example evaluation pipeline with Net2Brain using the dataset Michael F. Bonner et al. (2017; see main text). We perform feature extraction (fx), RDM creation (rdm), and RSA analysis 10 times and report the average. We compare the usage of CPU vs GPU on an AMD EPYC 7313 16-Core Processor and a single NVIDIA A100-SXM4-80GB. Note that RSA does not use the GPU, which is why its GPU memory usage is reported as 0.

		average time (s)			peak cpu memory (mb)			peak gpu memory (mb)		
	name	fx	rdm	rsa	fx	rdm	rsa	fx	rdm	rsa
[t]2*AlexNet	cpu	1.082	0.252	4.348	172.111	206.750	170.159	0	0	0
	cuda	0.699	0.196	4.329	139.917	141.209	138.081	773	220	0
[t]2*ResNet50	cpu	5.302	0.505	5.373	174.583	178.007	167.397	0	0	0
	cuda	1.473	0.357	5.350	147.380	152.401	143.304	933	734	0
[t]2*ViT/16-base	cpu	5.497	0.902	4.309	151.103	150.866	151.155	0	0	0
	cuda	1.384	0.713	4.358	124.341	135.485	142.516	1025	172	0

Table S3. Distribution of models by architecture type.

Architecture Type	Count	Example Architectures	Example Applications in Neuroscience
CNN	522	VGG16, ResNet50, EfficientNet	Visual cortex modeling, object recognition
Transformer	13	BERT, ALBERT, GPT	Attention allocation and working memory mechanisms
Vision Transformer	80	ViT-B/16, DeiT, CaiT	Global visual attention
Swin-Transformer	10	Swin-B, Swin-S, Swin-L	Multi-scale visual information processing in cortical hierarchies
MLP-Mixer	25	Mixer-B/16, ResMLP, gMixer	Alternative to convolutional processing
Multimodal	5	CLIP (RN50), CLIP (ViT-B/32)	Cross-modal integration

Table S4. Distribution of models by task category.

Task Category	Count	Example Architectures	Example Applications in Neuroscience
Image Classification	520	ResNet50, VGG16, DenseNet121	Ventral visual stream, object recognition
Object Detection	32	Faster R-CNN, YOLO, RetinaNet	Attentional selection and figure-ground mechanisms
Panoptic Segmentation	4	Panoptic-FPN (R50, R101)	Scene parsing, figure-ground segregation
Semantic Segmentation	1	SceneParsing	Scene parsing, figure-ground segregation
Instance Segmentation	24	Mask R-CNN, Cascade R-CNN	Object individuation and boundary detection processes
Keypoint Detection	4	Keypoint R-CNN (R50, R101)	Motion perception
Various Visual Tasks	23	Denoisng, Edge Occlusion	Visual processing across specialized cortical regions
Video Classification	6	SlowFast, X3D, Slow-R50	Motion processing, temporal integration
Natural Language Processing	6	Albert-Base-v2, GPT2	Language network processing
Audio Tagging	29	PANNs CNN10, PANNs ResNet38	Auditory processing pathways

Table S5. Available Regions of Interest (ROIs) in Net2Brain for each dataset.

Dataset	Regions of Interest (ROIs)
Algonauts 2019	EVC, IT, whole brain MEG
Algonauts 2021 (Bold Moments)	V1, V2, V3, V3ab, V4, 7AL, BA2, BMDgeneral, EBA, FFA, IPS0, IPS1-2-3, LOC, MT, OFA, PPA, PF, PFop, PFt, RSC, STS, TOS, whole brain, hemi-left, hemi-right
Algonauts 2023 (NSD-Subset)	V1, V2, V3, hV4, EBA, FBA-1, FBA-2, FFA-1, FFA-2, Mfs-words, OFA, OPA, OWFA, PPA, RSC, VWFA-1, VWFA-2, hemi-left, hemi-right
Algonauts 2023 (872 Subjects Subset)	V1d, V2d, V3d, V1v, V2v, V3v, hV4, EBA, FBA-1, FBA-2, FFA-1, FFA-2, Mfs-words, OFA, OPA, OWFA, PPA, RSC
THINGS-fMRI	V1, V2, V3, hV4, VO1, VO2, lTOS, rTOS, lLOC, rLOC, lFFA, rFFA, lPPA, rPPA
Bonner et al.	V1d, V1v, V2d, V2v, V3a, V3b, V3d, V3v, hV4, LO1, LO2, PHC1, PHC2, VO1, VO2
